# Identification of metastasis-associated microRNAs in serum from rectal cancer patients

**DOI:** 10.18632/oncotarget.21412

**Published:** 2017-09-30

**Authors:** Robin Mjelle, Kjersti Sellæg, Pål Sætrom, Liv Thommesen, Wenche Sjursen, Eva Hofsli

**Affiliations:** ^1^ Department of Clinical and Molecular Medicine, NO-7491 Trondheim, Norway; ^2^ Department of Computer Science, Norwegian University of Science and Technology, NO-7491 Trondheim, Norway; ^3^ Department of Biomedical Science, Norwegian University of Science and Technology, 7030 Trondheim Norway; ^4^ Department of Medical Genetics, St. Olavs Hospital, Norwegian University of Science and Technology, 7030 Trondheim Norway; ^5^ The Cancer Clinic, St. Olavs Hospital, Trondheim University Hospital, 7030 Trondheim, Norway

**Keywords:** rectal cancer, biomarker, microRNA, serum, isomiR

## Abstract

MicroRNAs (miRNAs) are promising prognostic and diagnostic biomarkers due to their high stability in blood. Here we investigate the expression of miRNAs and other noncoding (nc) RNAs in serum of rectal cancer patients. Serum from 96 rectal cancer patients was profiled using small RNA sequencing and expression of small RNAs was correlated with the clinicopathological characteristics of the patients. Multiple classes of RNAs were detected, including miRNAs and fragments of tRNAs, snoRNAs, long ncRNAs, and other classes of RNAs. Several miRNAs, miRNA variants (isomiRs) and other ncRNAs were differentially expressed between Stage IV and Stage I-III rectal cancer patients, including several members of the miR-320 family. Furthermore, we show that high expression of miR-320d as well as one tRNA fragment is associated with poor survival. We also show that several miRNAs and isomiRs are differentially expressed between patients receiving preoperative chemoradiotherapy and patients who did not receive any treatment before serum collection. In summary, our study shows that the expression of miRNAs and other small ncRNAs in serum may be used to predict distant metastasis and survival in rectal cancer.

## INTRODUCTION

About 15–25% of colorectal cancer (CRC) patients have distant metastasis at the time of diagnosis [1]. Only 44% of patients with rectal cancer are diagnosed when the disease is at a local stage, for which the 5-year survival rate is 90% [1]. The survival rate declines rapidly to 12% for patients diagnosed with distant metastasis [1]. The current prognostic and diagnostic biomarkers of rectal cancer are compromised by limited sensitivity and specificity [2] and there is therefore a need for new biomarkers.

MicroRNAs (miRNAs) and other small noncoding (nc) RNAs are promising biomarkers in cancer, and are easily detectable in serum. Only a few studies have investigated the association between circulating miRNAs and tumor staging in rectal cancer, and there is limited overlap between the results from these studies due to differences in methodology and study populations [3].

Our group has previously demonstrated that serum miRNAs are differentially expressed in serum of CRC patients compared to healthy subjects [4]. Whereas previous studies mainly used microarrays and qRT-PCR to assess the miRNA abundance, we here used High throughput sequencing (HTS) to create a comprehensive serum profile of small RNAs. We measured the expression of canonical miRNAs and miRNA variants (isomiRs), as well as other small ncRNAs.

Our analyses show that multiple classes of small RNAs are present in serum with potential prognostic value. We find that the expression levels of several miRNAs, isomiRs and other small ncRNAs are associated with disease stage. Specifically, we show that several members of the miR-320 family are up-regulated in metastatic patients and that high expression of miR-320d and a tRNA fragment is associated with poor survival. We also show that some miRNAs are differentially expressed between serum samples collected before pre-operative radiation treatment compared to samples collected after treatment.

To our knowledge, this is the most comprehensive profiling of circulating small ncRNAs in rectal cancer patients and this study demonstrates the potential of small ncRNAs as prognostic and diagnostic biomarkers.

## RESULTS

### Patient cohort characteristics

General information and relevant data collected from the patients’ medical records that were used in the results are presented in Table 1. Assessment of distant metastases, local recurrence, and tumor classification according to the 5^th^ edition of the TNM staging system, was performed with help from a surgeon and an experienced oncologist, respectively. The patient cohort was separated into two main groups, one group where serum was collected before preoperative treatment and one group where serum was collected after preoperative treatment (Table 1 and Supplementary Figure 1). To avoid potential confounding factors, analyses that compared clinicopathological groups were restricted to patients that did not receive treatment before serum sampling (n=53). A separate analysis was performed to investigate how treatment affects miRNA expression.

**Table 1:** Clinical and histopathological characteristics of the investigated patient cohort

	Variable	Number of patients
Gender		
	Male	53
	Female	43
Age at diagnosis		
	<39	3
	40-49	5
	50-59	19
	60-69	32
	70-79	19
	80-89	17
	>90	1
T-Stage		
	Tis	1
	T1	5
	T2	11
	T3	43
	T4	36
N-Stage^*^		
	N0	57
	N1	14
	N2	23
M-Stage		
	M0	75
	M1	21
Stage grouping		
	0	1
	I	13
	II	38
	III	23
	IV	21
KRAS mutation		
	Wild type	70
	Mutation	26
Serum collection		
	Before treatment	53
	After treatment	43

^*^N-stage of two patients could not be assessed.

### Small RNA sequencing

High throughput sequencing of small RNAs were performed on 96 samples with high quality of all samples (Supplementary Figure 2). On average, 10 964 086 reads mapped to the human genome per sample (Supplementary Figure 3A). To determine which types of RNA are expressed in serum we first annotated the sequences to the miRNA database miRBase v21 [5]. Sequences that did not match to any miRNA in miRBase were annotated to the RNA Central database of ncRNAs (http://rnacentral.org). The results from these two annotations showed that the sequencing libraries were dominated by small cytoplasmic RNAs (scRNAs), miRNAs and long non-coding RNAs (lncRNAs) (Supplementary Figure 3B). A smaller fraction of ribosomal RNAs (rRNAs), transfer RNAs (tRNAs) and small nucleolar RNAs (snoRNAs) were also detected (Supplementary Figure 3B). The length distribution of the sequences revealed three clear peaks around 13, 22 and 31 nucleotides (nts) (Supplementary Figure 3C). The 22 nt peak mainly consists of miRNAs, the 31 nt consists of snoRNAs, scRNAs and tRNAs, whereas the wide peak around 13 nts consists of fragments of the various RNA classes (Supplementary Figure 3D).

In total, 498 canonical miRNAs were detected in the sequencing experiment, 215 of which were expressed in all 96 samples. The highest expressed miRNA was miR-486-5p, with a mean counts per million (cpm) of 174 428 reads across all samples (Supplementary Figure 3E). Multidimensional scaling (MDS) of the mature miRNAs revealed no clear subgrouping of the samples (Supplementary Figure 3F) and the number of miRNA reads were relatively even across samples (Supplementary Figure 3B and 3G).

To normalize the expression of the detected RNAs, we used a set of 10 different 22 nts calibrator RNAs that were mixed with the patient RNA during library preparation (Supplementary Figure 4A). All calibrators were detected in the sequencing data as well as several variants of the same calibrators, which probably represents variations during RNA synthesis or random degradation of the RNA (Supplementary Figure 4B). No significant sample variation was detected for the calibrators, indicating that library preparation and sequencing performance were equal across samples (Supplementary Figure 4B and 4C).

Having determined the expression of the canonical miRNAs, we analyzed the expression of miRNA variants (isomiRs) (Supplementary Figure 4D). We detected 8757 unique isomiRs and after removing sequences containing mismatches to the genome, the number was reduced to 3758 unique isomiRs. The highest number of isomiRs was found for miR-486-5p with 105 unique isomiRs, followed by miR-320a with 94 isomiRs (Supplementary Table 1). When separating the main classes of isomiRs [6], we found that IsomiRs with 3’ trimming and non-templated additions (NTAs) were the most frequent isomiR type, comprising 32% and 39% of all unique isomiRs, respectively (Supplementary Table 2). Only 14% of the unique isomiRs had modifications at the 5’ end of the sequence.

Of the sequences that successfully matched to RNAs in the RNA Central database, 11563 were expressed in at least one of the samples, and 285 were expressed in all 96 samples. The ncRNAs were dominated by the Y-RNA scRNA-hY4_RNA (RNA Central ID: URS0000188F7D), which was expressed in multiple variants. The highest expressed variant was 32 nts long and had a mean expression of 537 021 cpm (Supplementary Table 3). In comparison, the highest expressed miRNAs, miR-486-5p, had a mean expression around 170 000 cpm (Supplementary Figure 3E).

### Multiple miRNAs and ncRNAs predict metastasis

**Figure 1:**
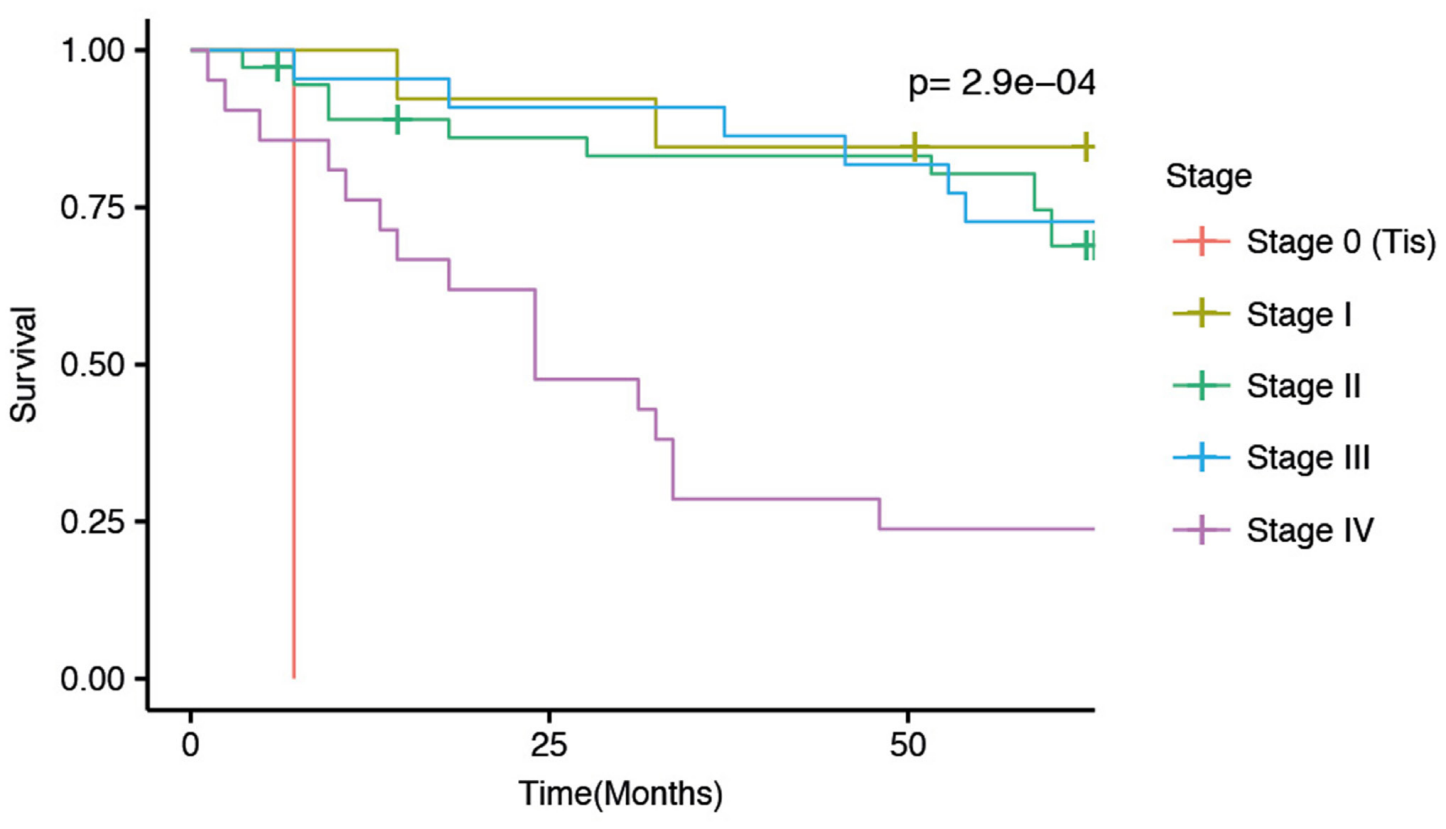
Kaplan-Meier 5-year survial curves for the current patient cohort separated by disease stage at diagnosis The p-value indicate differences in survival between the five stages.

**Figure 2:**
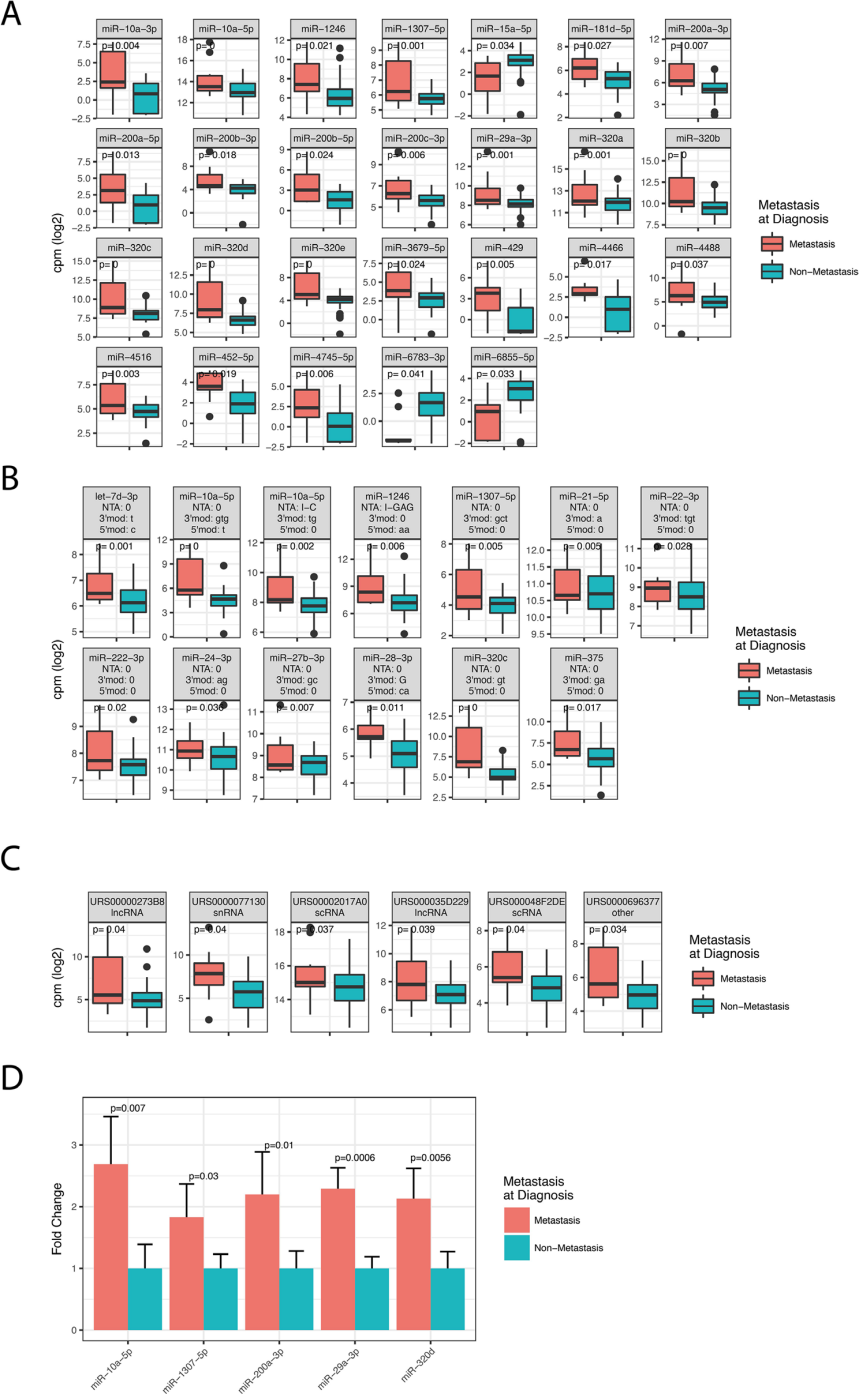
Differentially expressed miRNAs, isomiRs and ncRNAs between patients diagnosed with metastatic disease (stage IV) and non-metastatic disease (stage I-III) The figure shows box-plots of significant canonical miRNAs **(A)**, selected isomiRs **(B)**, and ncRNAs **(C)**. P-values in the plots are corrected for multiple testing with the Benjamini-Hochberg procedure. (B) IsomiR modification type is indicated above each facet as: NTA: Non-templated addition; 3’mod: Modifications at the three prime end (lower case indicate trimming, upper case indicate tailing); 5’mod: Modifications at the five prime end (lower case indicate trimming, upper case indicate tailing). (C) The RNA Central ID is given above the ncRNA name and can be browsed at www.rnacentral.org. **(D)** Validation of the sequencing results by qRT-PCR for five canonical miRNAs. Results are expressed as mean fold change comparing metastasis to non-metastasis samples.

Cancer metastasis is generally associated with poor survival, also in rectal cancer. Our patient cohort showed reduced survival of stage IV patients compared to Stage I-III patients (Figure 1). To investigate how metastasis affects miRNA expression we compared patients with metastasis at the time of diagnosis to patients without metastasis at the time of diagnosis. The analysis included miRNAs that were expressed with at least 1 cpm in more than 50% of the patients. We detected 26 significant miRNAs (Figure 2A) and 61 significant isomiRs (Figure 2B and Supplementary Table 4) that were differentially expressed between the two groups. This included members of the miR-320 and miR-200 family as well as the highly expressed miR-10a.

Having shown that several miRNAs were associated with metastasis, we analyzed sequences that matched to RNA species in the RNA Central database. For the analysis we required the RNAs to be expressed with at least 1 cpm in all 96 samples to reduce potential bias from the sample preparation and gel purification. We identified six ncRNAs to be differentially expressed between the two groups, including two lncRNAs, two scRNAs, one snRNA and one Y-RNA (Figure 2C).

### Validation of metastasis associated miRNAs using qRT-PCR

To validate the HTS results we performed qRT-PCR on the following canonical miRNAs: miR-10a-5p, miR-1307-5p, miR-200a-3p, miR-29a-3p, and miR-320d. These miRNAs were selected because they were highly expressed in serum and previously found to be dysregulated in serum of various cancers [7–11]. Internal normalization controls were selected based on expression levels and the results from the *Normfinder* algorithm in R [12] (See Methods). The miRNAs were validated using ten Stage I samples selected from the cohort included in the sequencing and ten independent Stage IV rectal samples provided by the local biobank Biobank1 [13]. We found all five miRNAs to be differentially expressed between metastatic and non-metastatic patients (Figure 2D). Thus, the qRT-PCR results agreed with the HTS data, confirming that the five selected miRNAs are indeed dysregulated in serum of metastatic patients.

### Preoperative treatment affects miRNA expression

Since the patient cohort was divided in two groups, one group that received preoperative treatment before serum collection and one group that did not receive treatment, we wanted to investigate if the treatment could affect miRNA expression.

**Figure 3:**
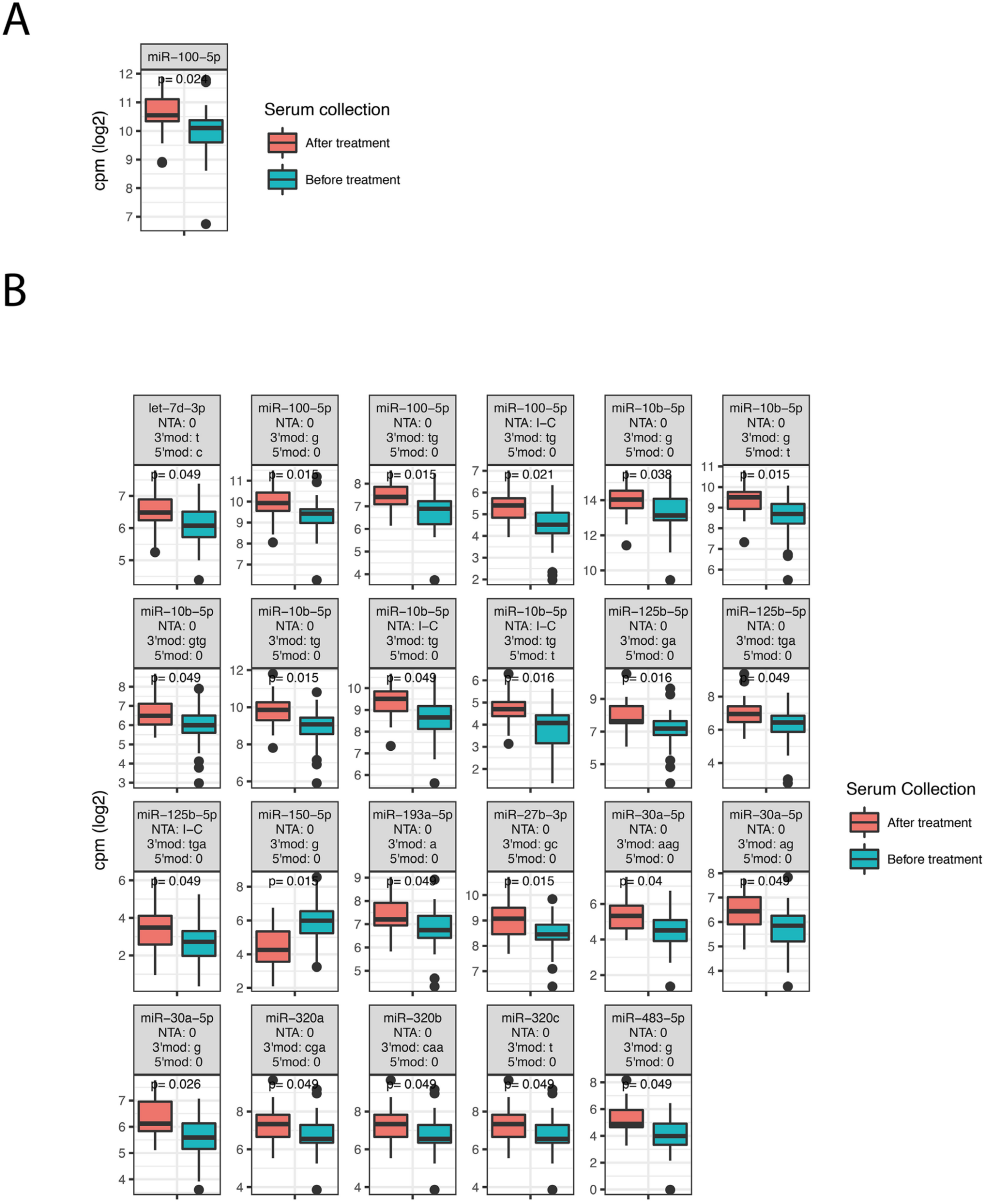
Differentially expressed miRNAs, isomiRs and ncRNAs between patients that received preoperative chemoradiotherapy before serum was collected and patients that did not receive any preoperative treatment The figure shows box-plots of significant canonical miRNAs **(A)** and isomiRs **(B)**. See Figure 2 for explanations of isomiR modification types.

The two groups consisted of 43 patients who received preoperative treatment before serum collection and 53 patients who did not receive any treatment before serum collection. The preoperative treatment group was filtered to only comprise patients receiving chemoradiotherapy (50 Gy/25 fractions and capecitabine twice daily (825 mg/m^2^)), while patients that underwent surgery (n=2), surgery and chemoradiotherapy (n=6) or surgery and adjuvant treatment (n=1) were excluded from the group. To investigate the effect of preoperative treatment on serum miRNAs we analyzed expression differences between the two groups. Patients with metastasis at diagnosis were also removed from this analysis, leaving 43 patients whose serum was collected before treatment and 27 patients whose serum was collected after treatment. The analysis required an expression of at least 1 cpm in more than 50% of the patients. We detected one miRNA, miR-100-5p, (Figure 3A) and 23 isomiRs (Figure 3B) that differed significantly between the two groups.

### Survival analysis

**Figure 4:**
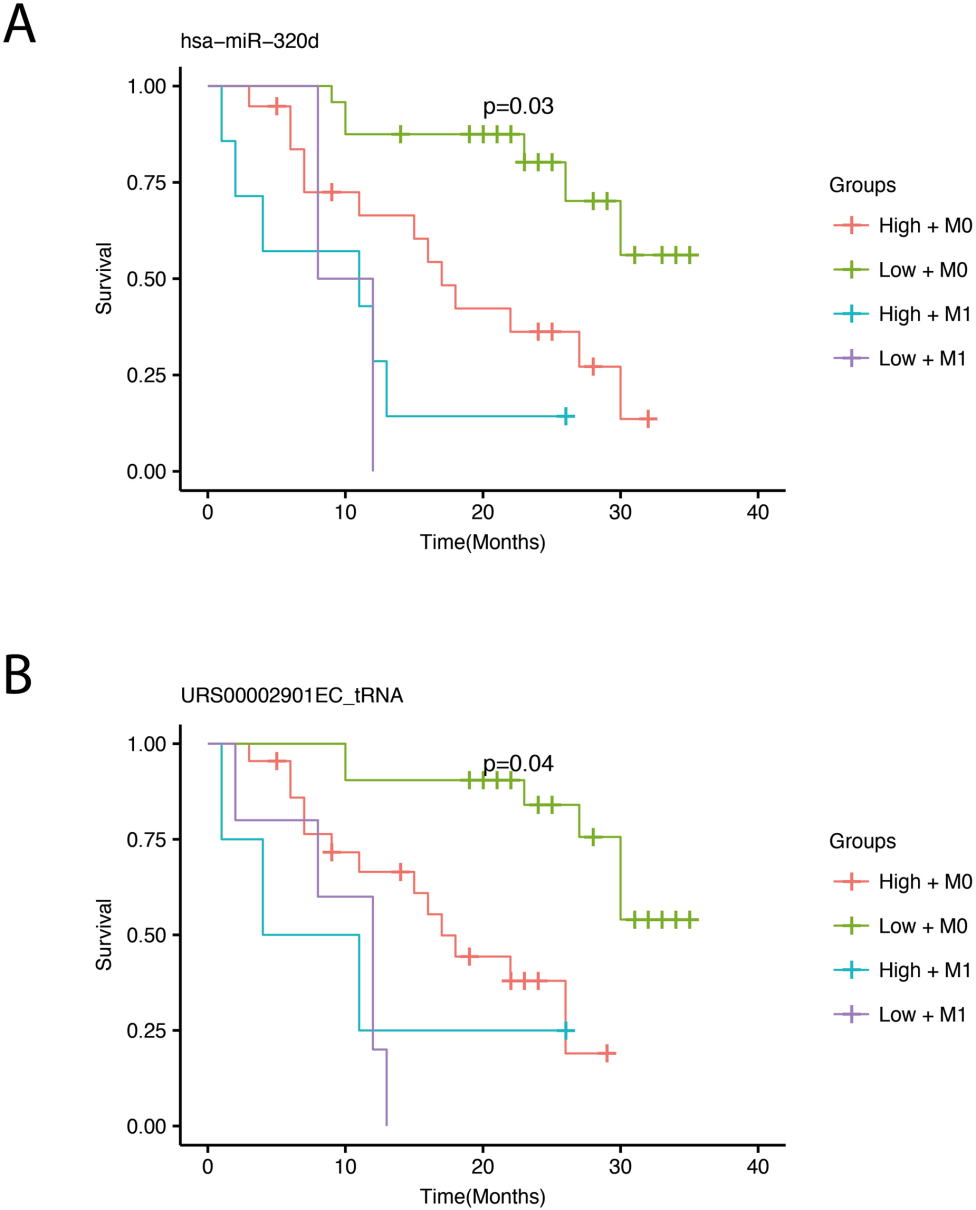
Survival plots showing overall survival with respect to expression adjusted for the presence of metastasis Survival curves for miR-320d **(A)** and the tRNA fragment **(B)**. The four survival curves represent high (red) and low (green) expression for patients without metastasis (M0) and high (turquoise) and low (violet) expression for patients with metastasis (M1). A cross on the survival curve indicates censoring. P-values in the plots are corrected for multiple testing using bonferroni correction and represents significance across the four groups. The RNA Central ID for the tRNA can be browsed at www.rnacentral.org.

We performed survival analysis by correlating miRNA expression with overall survival for the patients. The survival analyses were adjusted for the presence of distant metastasis at the time of diagnosis, since this factor correlates highly with survival (Figure 1). By using a conservative filtering approach, requiring more than 100 cpm in more than 50% of the samples, we detected one miRNA, miR-320d, and one tRNA fragment that significantly correlated with survival after adjusting for multiple testing (Figure 4). Low expression of both the miRNA and the tRNA was associated with better survival.

## DISCUSSION

Prognostic biomarkers that predict the spread of cancer to distant sites have important clinical applications. In our study population, 22% of the patients that did not have distant metastasis at the time of diagnosis developed metastasis at a later time. Some of these patients may have had metastasis that was not detected by imaging. To detect recurrence at an early time point good prediction methods are needed. The development of a blood-based biomarker will serve as an important invasive alternative to today's practice. In this study we demonstrate that the serum miRNA expression in patients with rectal cancer metastases is markedly different from that in patients with non-metastatic rectal cancer. Based on these findings we believe that circulating miRNAs and small ncRNAs are valuable prognostic biomarkers for rectal cancer and probably CRC as a group.

MiRNAs are closely linked to proliferation and differentiation and have been found to regulate specific steps in the metastatic pathway and in transforming tumor cells into metastatic malignancies [14–16]. Interestingly, Neerincx et al. found three of the same metastasis associated miRNAs as identified in our study (miR-300b, miR-300d and miR-1246) to be up-regulated in metastatic CRC tissue [17]. The same three miRNAs are proposed to have a role in CRC metastases formation, although the exact mechanism is still not clear [18–21]. CRC cells are found to actively secrete miR-1246 in microvesicles to promote angiogenesis by activating signaling pathways [20]. MiR-320b is found to be up-regulated in CRC with liver metastasis [21] and positively regulate the expression of metastasis promoting genes [21]. MiR-320d is highly expressed in the proliferative compartment of the colonic crypts of normal colonic mucosa in CRC [18]. Moreover, Hofsli et al. found serum miR-320a to be up-regulated in stage IV CRC compared to Stage I-II [4]. Of note, the study by Hofsli et al. used serum samples from the same biobank as the sequencing samples in the current study. The miR-200 family also plays a role in metastasis by promoting metastatic colonization by regulating the tumor cell secretome in breast cancer [22]. Overexpression of these miRNAs were shown to increase lung-colonization ability of poorly metastatic cancer cell lines and reduce tumor cell entry into circulation from primary tumors of mice, potentially by inhibiting epithelial–mesenchymal transition (EMT) [22]. Furthermore, *in situ* hybridization staining in CRC tissue revealed high expression of miR-200c in liver metastasized CRC tissues compared with adjacent hepatocytes [23]. Together, the literature supports that the miRNAs identified in the current study could be related to a metastatic phenotype.

In addition to miRNAs, several groups now investigate if other ncRNAs could be used as biomarkers in cancer. Since many of the changes in gene expression are reportedly related to epigenetic alterations, any transcript could potentially be affected [24]. Y-RNAs are shown to be functionally required for chromosomal DNA replication in mammalian cell nuclei [25]. Y-RNAs are frequently up-regulated in tumors and knock-down experiments in human cell lines have resulted in inhibition of cell proliferation [26]. Moreover, Y-RNAs have been detected in vesicles released by mouse immune cells [27], and are abundant in human serum and plasma. Although the function of Y-RNA fragments is not known, they may take part in cell signaling [28, 29], however, it remains to be proven if such signaling actually exists and what role it plays [29–32]. Recently, this class of RNAs were also shown to be expressed in serum and up-regulated in serum of breast cancer patients [33]. In our study, we report several Y-RNA fragments, previously not reported in the literature, to be up-regulated in serum of metastatic patients, in agreement with previous publications analyzing tissue and serum.

We analyzed miRNA expression differences between serum collected before and after preoperative chemoradiotherapy. Although this comparison does not include samples from the same patients before and after treatment, it indicates that miRNA expression does change as a response to chemoradiotherapy. Using formalin-fixed paraffin-embedded (FFPE) biopsies from locally advanced rectal cancer, Eriksen et al. identified two miRNAs in response to preoperative chemoradiotherapy [34].

The miRNA miR-30a-5p, here found to be up-regulated after treatment, was also found to be up-regulated in prostate cancer cells after radiation treatment [35]. Previous studies have shown that miRNAs do respond to treatment in CRC [36–40]. In a study by Dinh et al, they analyzed miRNA expression in plasma of lung cancer patients undergoing radiotherapy (RT) and found that levels of miR-150-5p and miR-29a-3p were inversely correlated with increasing RT dose. They also found that levels of miR-125b-5p increased with increased RT dose. The potential source of circulating miRNAs was investigated by measuring intra - and extracellular levels (exosomes) of miR-150-5p and miR-29a-3p in irradiated normal and lung cancer cells. They showed that miR-150-5p and miR-29a-3p levels were decreased in exosomes secreted by irradiated cells while intracellular expression increased upon radiation [41]. Decreased levels of miR-150 have also been found in whole blood of mice exposed to ionizing radiation [42]. To validate the treatment-associated miRNAs identified in the current study, patient-matched samples collected before and after treatment should be used.

## MATERIALS AND METHODS

### Patient samples

Patients included in this study were initially recruited from two Norwegian hospitals (St.Olavs Hospital and Hamar Hospital) between January 2007 and June 2008. Patient samples were stored at -80°C in a research Biobank after collection. Molecular characterization was performed on paraffin-embedded tumor specimens from the patients, and included Microsatellite (MSI) markers, mutation analyses of the oncogenes BRAF (V600E) and KRAS (exon 2 and 3), and methylation analyses of Mismatch Repair genes associated with Lynch Syndrome. Informed written consent was obtained from each patient, and the study was approved by The Regional Committee for Medical and Health Research Ethics in medical research and The National Data Inspectorate [43, 44]. In the initial cohort of CRC patients, 132 were diagnosed with rectal and rectosigmoid cancer. From the patients diagnosed at St.Olavs Hospital (n=102), 96 patients were randomly included in this study. Relevant data was collected from patient medical records and from The Norwegian Cancer Registry. The samples in the validation cohort were provided by Biobank1 [13]. The complete patient data is available in Supplementary Table 10.

### RNA isolation

Total RNA was isolated from 200μl patient serum using the QIAGEN miRNeasy serum/plasma kit. Briefly, QIAzol lysis buffer (1000μl) was added to the sample to stabilize the RNA by eliminating ribonucleases, cellular DNA and proteins released by cell lysis. Addition of chloroform (200μl) and subsequent centrifugation allowed phase separation of the lysate, and the upper aqueous supernatant was separated and mixed with ethanol before loaded onto the membrane in the spin column provided in the kit. RNA then binds to the column and contaminants were washed away before RNA was eluted using RNase-free water. Isolated RNA was stored at -80 °C.

### RNA quantification and quality assessment of isolated RNA

Isolated RNA was measured using NanoDrop ™ ND-1000 spectrophotometer to give an indication on RNA purity and concentration. For further assessment of RNA quality and relative size, a few randomly selected samples was measured using Eukaryote total RNA pico assay on the 2100 Bioanalyzer. Results showed that small RNAs were present in the samples at acceptable concentrations to continue the library preparation. It was assumed that the results of these samples were representative for all RNA samples. For total RNA assays, a ribosomal RNA ratio is determined giving an indication on RNA integrity. Ribosomal RNA (rRNA) is not expected to be present in cell-free serum, so the typical rRNA (ribosomal RNA) 28S:18S ratio and RNA integrity number (RIN) is not applicable.

### Preparation of cDNA library for small RNA sequencing

Small RNA sample preparation was performed using NEBNext^®^ Multiplex Small RNA Library prep set for Illumina (Set 1) according to the manufacturer's instructions. Briefly, 3’ and 5’ adaptors were sequentially ligated to serum total RNA, using 6μl input RNA per sample. A mix of ten different calibrator oligoribonucleotides (0.25μl) with known sequence and concentration were added in the 3’ligation step and used as internal standards as described by Hafner and colleagues [45]. The following steps included reverse transcription of the ligated fragments, amplification by PCR for 13 cycles using Index primers from NEBNext^®^ Multiplex Small RNA Library prep set for Illumina Set 1 and Set 2, and gel purification. Quality controls of the cDNA libraries were measured using High Sensitivity DNA assay on 2100 Bioanalyzer. The miRNA fragments were sequenced on the Illumina HiSeq system using 50 base pair single read at the Genomics Core Facility (GCF) at the Norwegian University of Science and Technology in Trondheim, Norway.

### Processing of sequence data

Quality control of the raw sequence data was performed using fastQC [46]. Trimming of sequence adapters from the 3’end of the raw sequences was performed using cutadapt-1.2.1 [47]. The trimmed sequences were collapsed with the fastx collapser tool (http://hannonlab.cshl.edu/fastx_toolkit/) into single unique reads along with their total read count and mapped to the human (hg38) genome using bowtie2 [48], allowing for up to 10 alignments per read to account for reads from duplicated miRNA loci (bowtie2 – k10). Reads overlapping with mature miRNA loci were identified using htseq-count from the HTseq python package [49]. These reads were further filtered to identify those with perfect alignment to the genome, and the total read count for mature miRNAs were then computed by summing the total read count per sequence (isomiR) overlapping each miRNA locus. Mature miRNAs and non-coding RNAs were annotated using miRBase (Release 21, 2014) and RNA Central (http://rnacentral.org) respectively. IsomiR variants were detected using SeqBuster [50] combined with a panel of in-house perl and R-scripts, which are available upon request. IsomiRs with mismatches to the genome were discarded from the analysis, as these could not be excluded as sequencing errors. However, isomiRs with non-templated addition at the 3’end were included in the analysis. Differentially expressed miRNAs and isomiRs were identified using the Bioconductor package limma combined with voom transformation [51, 52]. All miRNA sequence information was retrieved from miRBase [53]. In order to compare miRNA expression between samples, read counts were normalized using the calibrator RNA normalization factors calculated in limma, followed by counts per million (cpm) normalization. The calibrator RNAs were not filtered prior to normalization and the *calcNormFactors* in *limma* were calculated using the full calibrator count matrix. The processed count data is available in Supplementary Tables 6-9.

### Survival analysis

The survival analyses were performed using the R package *survival* and the *coxph* function. The parameters age and sex were included as covariates in all survival analysis, and metastasis status was included in the survival analysis for the ncRNAs. The p-values were adjusted for multiple testing by using bonferroni correction.

### Quantitative real-time PCR

Internal normalization controls for the qRT-PCR experiment were selected based on the criteria that they should be highly expressed in serum and not differentially expressed between metastatic and non-metastatic patients. By manually inspecting the *limma* results from the comparisons between metastatic and non-metastatic patients, we selected three miRNA as internal controls: miR-128a-3p, miR-92a-3p and miR-151a-3p. Further, we ran the normalization algorithm *Normfinder* on the HTS data to confirm that the three miRNA we selected indeed showed low variation and high stability across samples and between the metastatic and non-metastatic groups (see Supplementary Table 5 for complete results from *Normfinder*). The three miRNAs miR-128a-3p, miR-92a-3p and miR-151a-3p showed a group difference value of 0.04, 0.09 and 0.01, respectively, group standard deviation of 0.28, 0.55 and 0.47, and a stability value of 0.09, 0.15 and 0.12. In comparison, the median values for all miRNAs were 0.2 for group difference, 0.73 for group standard deviation and 0.235 for stability. From this we concluded that the three miRNAs were suited as internal normalization controls.

CDNA synthesis was performed using Applied Biosystems TaqMan Advanced miRNA cDNA Synthesis Kit from RNA isolated from 200 uL serum. The cDNA was diluted 1:10. The qPCR were performed on the StepOne Real-Time PCR System using TaqMan Advanced miRNA Assays following the manufacturer's Instructions. For miR-320d, the previous TaqMan cDNA and qPCR kit was used, as the advanced kit was not available. The miRNA expression data analysis determined by RT-qPCR was compared between metastasis and non-metastasis patients using unpaired Student's t-tests. ΔCt values were calculated by normalizing to the geometric mean of the internal control miRNAs. Relative quantities (RQ) were calculated as 2^–ΔCt^ and the RQ of the miRNAs of interest in each sample was determined as the mean RQ in the cDNA synthesis duplicates. All qRT-PCR experiments were performed in triplicates.

## SUPPLEMENTARY MATERIALS FIGURES AND TABLES




