# Supplementary material for: Identification of metastasis-associated microRNAs in serum from rectal cancer patients
Source: Oncotarget. 2017 Sep 30;8(52):90077–89. doi: 10.18632/oncotarget.21412 (PMC5685733; doi:10.18632/oncotarget.21412)
Supplement: Supplementary file 1 [file oncotarget-08-90077-s001.pdf]

Identification of metastasis-associated microRNAs in serum from rectal cancer patients

SUPPLEMENTARY MATERIALS

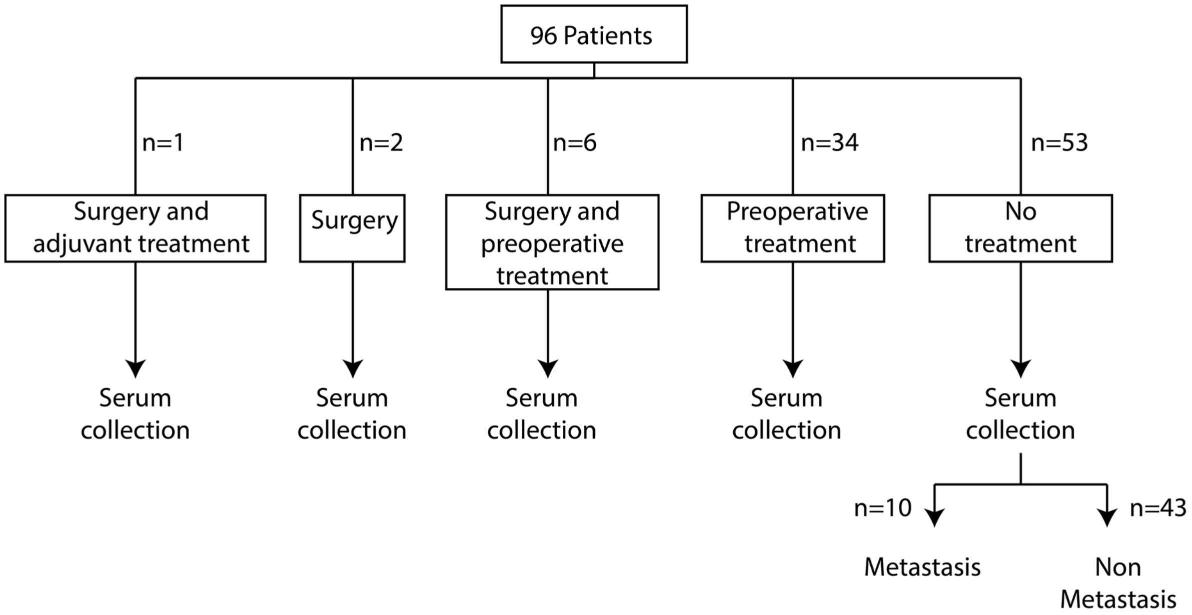

Supplementary Figure 1: Flow chart showing time of serum collection relative to treatment. For 53 patients the serum was collected prior to any treatment. For 43 patients, one or several types of treatments had been performed before the serum was collected.

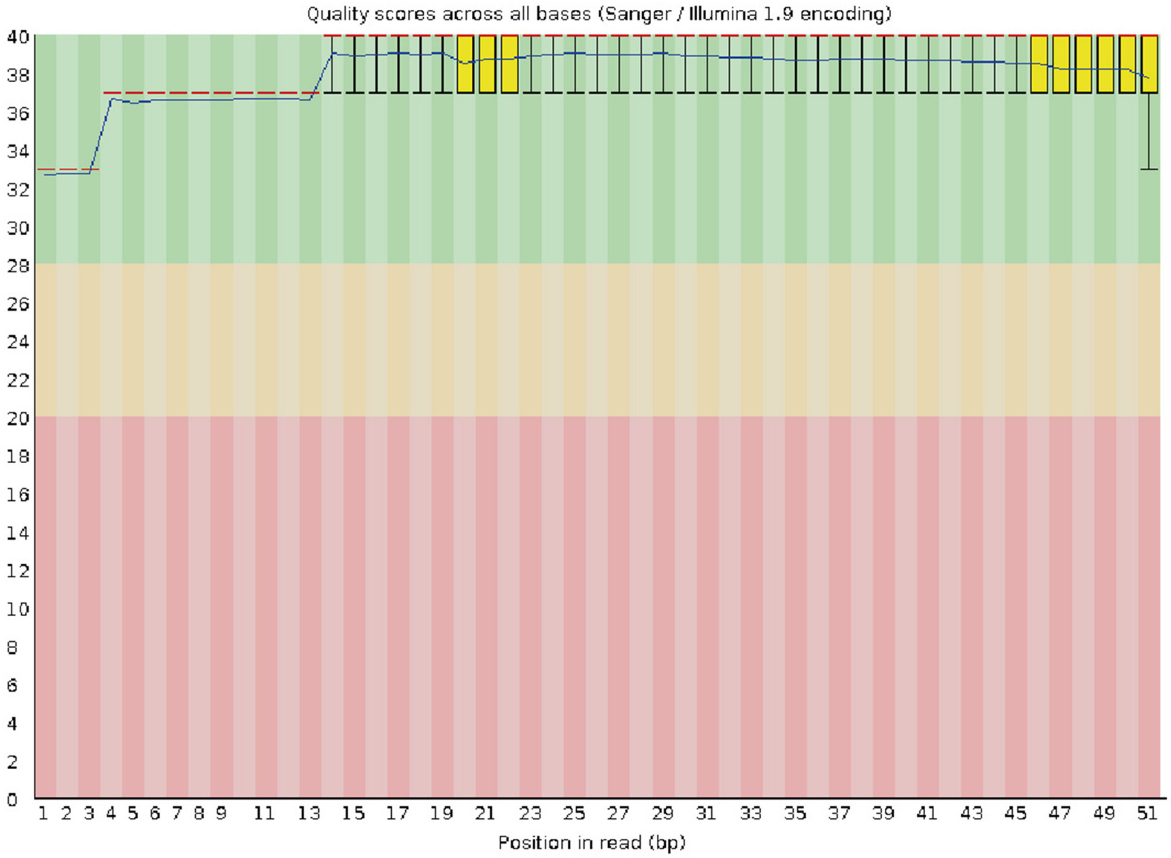

Supplementary Figure 2: Sequencing quality statistics from fastQC [54]. Quality score across all bases in the 51 base pair sequencing reads. This plot is representative of all the 96 samples.

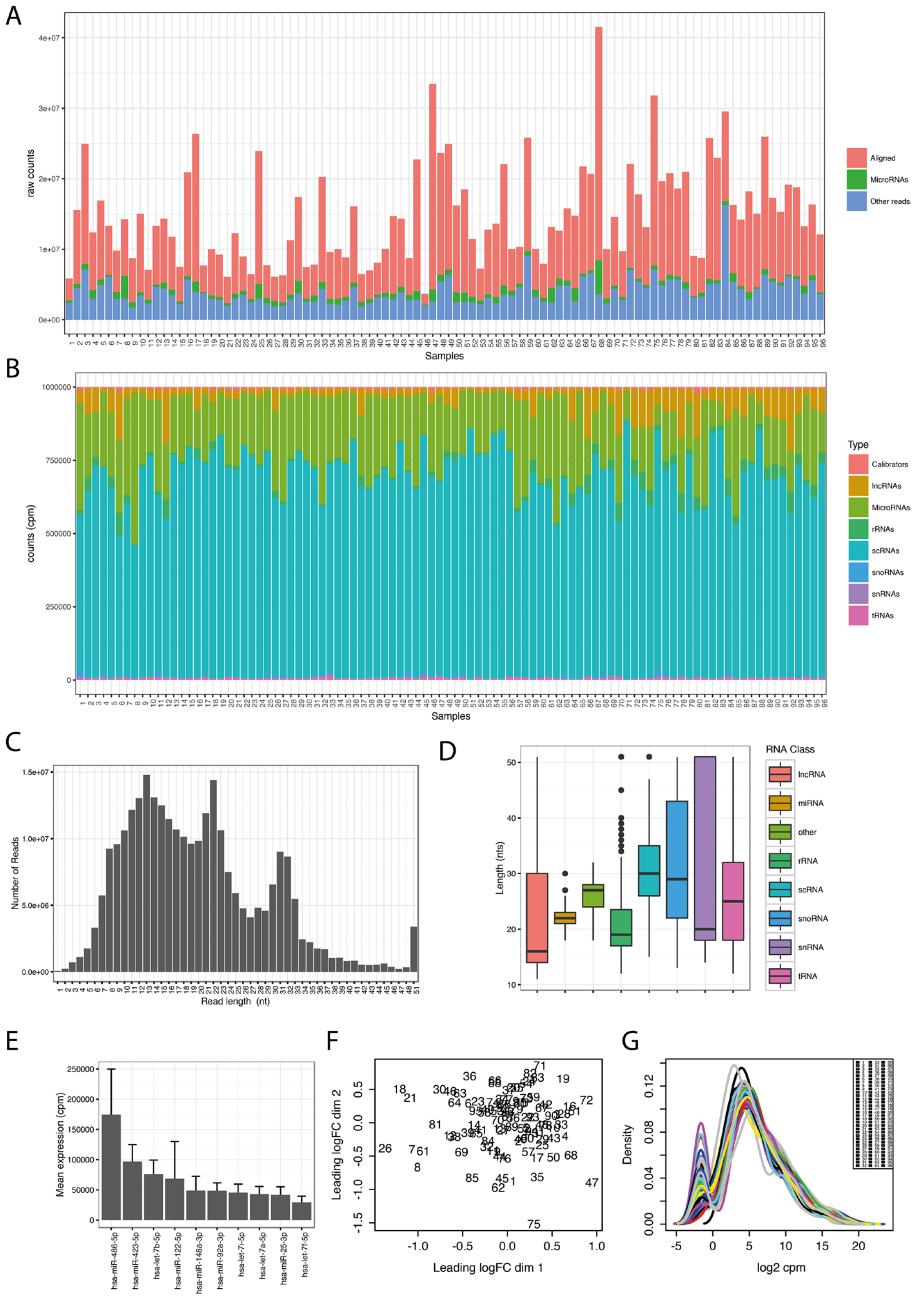

Supplementary Figure 3: Sequencing statistics. (A) Number of reads mapping to the human genome. Red: Aligned reads; Green: miRNA reads; Blue: other reads not contained in the two other classes. **(B)** Classes of aligned reads. Reads that successfully mapped to the genome were divided into the main RNA classes. **(C)** Length distribution of the sequence reads after adapter and quality trimming in 96 samples. The *x*-axis depicts the length of the sequence reads in nucleotides. The *y*-axis depicts the summed number of raw reads (counts per million (cpm)). The bars represent the mean read count per length. Abbreviations: nt, nucleotides. **(D)** Length distribution of the RNA classes based on the sequencing reads. **(E)** Mean expression of the top 10 expressed miRNAs. Error bars represent standard deviations across samples. **(F)** Multidimensional scaling (MDS) plot of normalized miRNA expression in 96 samples. The MDS plot was created using the MDS function in *limma* in R. The x-axis shows dimension 1 and the y-axis dimension 2. The samples are normalized using cpm normalization followed by normalization onto calibrator RNAs. Specifically the calibrator RNA normalization was performed by multiplying the cpm normalized matrix by the normalization-factors from the calcNormFactors function in the *edgeR* package in R. **(G)** Density plot of calibrator RNA normalized mature miRNAs. The figure shows the density of counts for the different samples. Overlapping densities indicate that the samples express approximately the same number and abundance of miRNA.

A

Calibrator ID	Sequence
Cal01	GTCCCACTCCGTAGATCTGTTC
Cal02	GATGTAACGAGTTGGAATGCAA
Cal03	TAGCATATCGAGCCTGAGAACA
Cal04	CATCGGTCGAACTTATGTGAAA
Cal05	GAAGCACATTCGCACATCATAT
Cal06	TCTTAACCCGGACCAGAAACTA
Cal07	AGGTTCCGGATAAGTAAGAGCC
Cal08	TAATCTCCTTAAGCGAATCTCGC
Cal09	AAAGTAGCATCCGAAATACGGA
Cal10	TGATACGGATGTTATACGCAGC

B

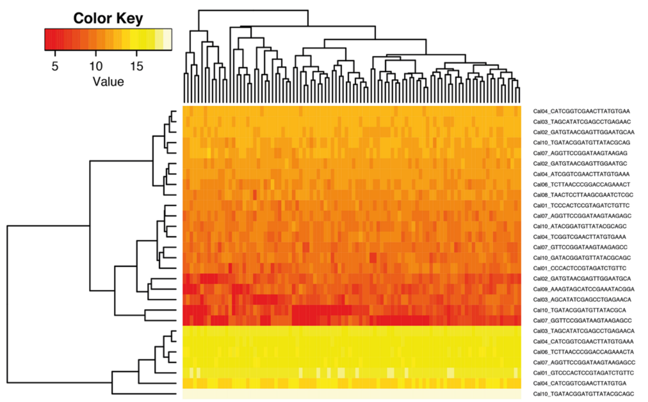

C

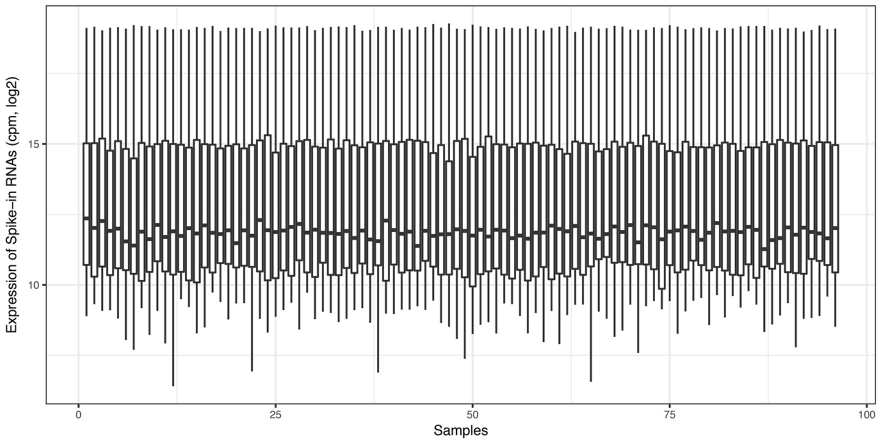

D

3' non-tempated addition	TACCCTGTAGAACCGAATTTGTGTA
3' tailing	TACCCTGTAGAACCGAATTTGTGTG
3' trimming	TACCCTGTAGAACCGAATTTGTG
5' tailing	ATACCCTGTAGAACCGAATTTGTGT
5' trimming	ACCCTGTAGAACCGAATTTGTGT
canonical hsa-miR-10b-5p	TACCCTGTAGAACCGAATTTGTGT
precursor hsa-miR-10b-5p	TATATACCCTGTAGAACCGAATTTGTGTGGTATCCGTATAGTCACAGATTCGATTCTAGGGGAATATA

Supplementary Figure 4: Overview of calibrator RNA and isomiR types. (A) Sequence of the calibrator RNAs used in the study. **(B)** Heat map of normalized calibrator RNAs (cpm, log2). Both the canonical calibrator RNA sequence (as listed in A) and shorter variants are shown. Yellow colour indicates high expression and dark red indicate low expression. **(C)** Boxplot of normalized calibrator RNAs (cpm, log2) as illustrated in B). **(D)** Illustrations of the main classes of isomiRs analyzed in the current study, exemplified for hsa-miR-10b-5p.

Supplementary Table 1: Number of isomiRs for the canonical miRNAs. The "miRNA" column lists the canonical mature miRNA name (from miRBase 21.0) and the "Number of isomiRs" column lists the number of unique isomiR sequences detected for the corresponding mature miRNA locus.

See Supplementary File 1

Supplementary Table 2: Number of isomiRs of the different types.

IsomiR type	Number of isomiRs
3'end tailing	766
3'end trimming	1897
5'end tailing	272
5'end trimming	543
Non templated additions (NTAs)	2296
Mismatch	4999

The “IsomiR type” column lists the classes of isomiRs analysed (see Supplementary Figure 4D) and “Number of isomiRs” lists the correspinding number of unique isomiR sequences.

Supplementary Table 3: Expression values (cpm) for ncRNAs in the RNA Central database. The RNAs (listed in the "Non coding RNA" column) are annotated with RNA Central ID, RNA type, and the detected sequence (ID and type are separated by "-"; type and sequence are separated by "_").

See Supplementary File 1

Supplementary Table 4: Differentially expessed IsomiRs between M0 (non-metastasis) and M1 (metastasis) patients at diagnosis. The statistical comparison used was M1-M0 such that a positive "Fold Change" indicates that the corresponding miRNA is up-regulated in M1 compared to M0. "miRNA" lists the canonical mature miRNA name (from miRBase 21.0); "Sequence" lists the specific isomiR sequence; "NTA" shows whether the isomiR contains non-template additions (NTAs) in the 3' end ("0" for no NTAs; "I-" followed by the specific NTA sequence, otherwise); "5' mod" and "3' mod" show whether the isomiR's 5' and 3' end, respectively, are identical to (0) or different from the canonical miRBase mature sequence (with lower and upper case letters representing, respectively, loss and addition of the corresponding nucleotides); "Fold Change (Log2)" is the log2 fold change of the M1-M0 statistical comparison (corresponding to the log2 of the isomiR's average expression in M1 divided by its average expression in M0; values computed by limma); "Average Expression" is the isomiRs average log2 cpm expression in the dataset (as computed by limma); "Adjusted P-value" is the Benjamini-Hochberg adjusted p-value.

See Supplementary File 1

Supplementary Table 5: Results from the *Normfinder* algorithmn. Selected internal controls are shown in bold. "miRNA" is the canonical mature miRNA name (from miRBase 21.0); "GroupDif" is the estimated difference in expression between the metastatic and non-metastatic group; "GroupSD" is a weighted average of the estimated intra-group variation; "Stability" is the *Normfinder* expression stability measure, where lower values mean more stable gene expression. See the *Normfinder* documentation (https://moma.dk/normfinder-software) and Andersen et al. (https://doi.org/10.1158/0008-5472.CAN-04-0496) for additional details.

See Supplementary File 1

Supplementary Table 6: Count matrix for canonical miRNAs. The count values are un-normalized. The column names represent sample ID.

See Supplementary File 1

Supplementary Table 7: Count matrix for isomiRs: The isomiRs (listed in the "IsomiR" column) are annotated with miRNA name and the detected sequence, separated by "_". The matrix shows isomiRs that were expressed in all 96 samples. The count values are un-normalized. The column names represent sample ID.

See Supplementary File 1

Supplementary Table 8: Count matrix for ncRNAs. The RNAs (listed in the "Non coding RNA" column) are annotated with RNA Central ID, RNA type, and the detected sequence (ID and type are separated by "-"; type and sequence are separated by "_"). The matrix shows ncRNAs that were expressed in all 96 samples. The count values are un-normalized. The column names represent sample ID.

See Supplementary File 1

Supplementary Table 9: Count matrix for calibrator RNAs. The calibrators (listed in the “Calibrator RNA” column) are annotated with calibrator ID and the detected sequence, separated by “_”. The count values are un-normalized. The column names represent sample ID.

See Supplementary File 1

Supplementary Table 10: Clinicopathological characteristics of the patients included in the current study

See Supplementary File 1
